# Effect of S-1 Plus Oxaliplatin Compared With Fluorouracil, Leucovorin Plus Oxaliplatin as Perioperative Chemotherapy for Locally Advanced, Resectable Gastric Cancer: A Randomized Clinical Trial

**DOI:** 10.1001/jamanetworkopen.2022.0426

**Published:** 2022-02-28

**Authors:** Jiren Yu, Yuan Gao, Li Chen, Dan Wu, Qianyun Shen, Zhicheng Zhao, Weihuai Liu, Hanliang Yang, Qi Zhang, Xinbao Wang, Ping Hu, Zhiqiang Zheng, Xianfa Wang, Hongjun Liu, Zekuan Xu, Zhilong Yan, Yingjie Wu, Mingjuan Jin, Qing Zhang, Xiaosun Liu, Kankai Zhu, Chunhui Shou

**Affiliations:** 1 Department of Gastrointestinal Surgery, The First Affiliated Hospital, Zhejiang University School of Medicine, Hangzhou, China; 2 Department of Gastrointestinal Surgery, The Second Affiliated Hospital, Zhejiang University School of Medicine, Hangzhou, China; 3 Department of General Surgery, Beilun District People’s Hospital, Ningbo, China; 4 Department of Gastrointestinal Surgery, Zhejiang Provincial Hospital of Chinese Medicine, Hangzhou, China; 5 Department of Hepatopancreatobiliary Surgery, Cancer Hospital of the University of Chinese Academy of Sciences, Hangzhou, China; 6 Department of General Surgery, The Central Hospital of Lishui City, Lishui, China; 7 Department of Gastrointestinal Surgery, The Second Affiliated Hospital of Wenzhou Medical College, Wenzhou, China; 8 Department of General Surgery, The Sir Run Run Shaw Hospital, Medical School Zhejiang University, Hangzhou, China; 9 Department of Gastrointestinal Surgery, Shandong Provincial Hospital, Jinan, China; 10 Department of General Surgery, The First Affiliated Hospital of Nanjing Medical University, Nanjing, China; 11 Department of Gastrointestinal Surgery, Ningbo First Hospital, Ningbo, China; 12 Department of Thyroid and Breast Surgery, The Affiliated People’s Hospital of Ningbo University, Ningbo, China; 13 Department of Epidemiology and Biostatistics, School of Public Health, Zhejiang University, Hangzhou, China

## Abstract

**Importance:**

Perioperative chemotherapy is a potential treatment for locally advanced gastric cancer. However, the optimal chemotherapy regimen remains unknown.

**Objective:**

To investigate the safety and efficacy of S-1 plus oxaliplatin (SOX) vs fluorouracil, leucovorin, and oxaliplatin (FOLFOX) as a perioperative chemotherapy regimen for patients with locally advanced gastric cancer.

**Design, Setting, and Participants:**

In this phase 3, open-label, multicenter, randomized clinical trial, patients from 12 Chinese hospitals were enrolled between June 2011 and August 2016, with a last follow-up date of September 2019. The primary tumor was evaluated as either invading the serosa or the adjacent structures with or without metastatic lymph nodes, and with no evidence of distant metastases. Data were analyzed from December 2019 to June 2020.

**Interventions:**

Patients were randomly assigned (1:1) to receive either 6 perioperative (2-4 preoperative and 2-4 postoperative) 3-week cycles of 130 mg/m^2^ oxaliplatin on day 1 and 80 to 120 mg/d S-1 orally daily for 2 weeks (SOX) or 130 mg/m^2^ oxaliplatin, 400 mg/m^2^ fluorouracil, 400 mg/m^2^ leucovorin, and 2400 mg/m^2^ fluorouracil as 46-hour infusion on day 1 (FOLFOX).

**Main Outcomes and Measures:**

The primary end point was 3-year overall survival (OS). An absolute noninferiority margin of –8% was chosen.

**Results:**

A total of 583 patients were enrolled; 293 were randomized to the SOX group and 290 were randomized to the FOLFOX group. Twelve patients (2.1%) refused preoperative chemotherapy (5 patients in the SOX group and 7 patients in the FOLFOX group), leaving a total of 288 patients in the SOX group (median [range] age, 61 [24 to 78] years; 197 men [68.4%]) and 283 patients in the FOLFOX group (median [range] age, 62 [24 to 80] years; 209 men [73.9%]) who received preoperative chemotherapy. The 3-year OS rate was 75.2% (95% CI, 70.3% to 80.5%) in the SOX group and 67.8% (95% CI, 62.5% to 73.5%) in the FOLFOX group. The absolute difference of 3-year OS rate between the 2 groups was 7.4% (95% CI, −0.1% to 14.9%), which was greater than the prespecified noninferiority margin (–8%) and showed the noninferiority of perioperative chemotherapy with SOX compared with FOLFOX.

**Conclusions and Relevance:**

In this randomized clinical trial, SOX was noninferior to FOLFOX as perioperative chemotherapy for patients with locally advanced gastric cancer and could be recommended as an alternative treatment for these patients in Asia.

**Trial Registration:**

ClinicalTrials.gov Identifier: NCT01364376

## Key Points

**Question:**

Is S-1 plus oxaliplatin (SOX) safe and effective as a perioperative chemotherapy regimen for patients with locally advanced gastric cancer?

**Findings:**

A total of 583 patients were enrolled in this phase 3 randomized clinical trial. SOX was noninferior to fluorouracil, leucovorin plus oxaliplatin as perioperative chemotherapy regimen for patients with locally advanced gastric cancer.

**Meaning:**

These findings suggest that SOX could be recommended as an alternative perioperative treatment for patients with locally advanced gastric cancer in Asia.

## Teaser

This randomized clinical trial investigates the safety and efficacy of S-1 plus oxaliplatin vs fluorouracil, leucovorin plus oxaliplatin as a perioperative chemotherapy regimen for patients with locally advanced gastric cancer.

## Introduction

Gastric cancer is the fifth most common malignant neoplasm and the third leading cause of cancer-related death worldwide.^1^ The prognosis remains poor for patients with advanced gastric cancer, especially when the primary tumor penetrates the serosa or invades the surrounding structures, and the 5-year survival rate for these patients is approximately 25%.^2,3^ Therefore, multimodal treatment is necessary to improve the prognosis of patients with advanced gastric cancer.

Perioperative chemotherapy has been shown to improve the overall survival (OS) of patients with resectable gastric cancer compared with surgery alone in 2 phase 3 randomized clinical trials (RCTs).^4,5^ One of the RCTs demonstrated that perioperative chemotherapy with a triplet regimen of epirubicin, cisplatin, and fluorouracil improved survival.^4^ The other study also showed a significant increase of 5-year OS in patients who received perioperative chemotherapy with fluorouracil and cisplatin.^5^ In spite of these advances, the prognosis of patients with locally advanced, resectable gastric cancer remains unsatisfactory.

S-1 (TS-1; Taiho Pharmaceutical) is an oral anticancer drug that combines tegafur (a prodrug of fluorouracil), gimeracil (an inhibitor of dihydropyrimidine dehydrogenase), and potassium oteracil (an inhibitor of the phosphorylation of fluorouracil in the gastrointestinal tract) in a molar ratio of 1:0.4:1.^6^ S-1 monotherapy has been shown to be an effective adjuvant treatment for advanced gastric cancer after radical resection.^7^ It has been reported that S-1 is noninferior to infusion fluorouracil in unresectable or recurrent gastric cancer.^8^ Although cisplatin plus S-1 did not demonstrate superiority to cisplatin plus infusion fluorouracil in Western countries, S-1 could be substituted for infusion fluorouracil for a more convenient administration.^8^ Given that the clinical response of S-1 combined with oxaliplatin (SOX) for unresectable or recurrent gastric adenocarcinoma ranged from 55% to 59%, with a favorable hematological toxic effects profile,^9,10^ it is reasonable that SOX could be an appropriate candidate regimen as perioperative chemotherapy for advanced gastric cancer. Therefore, we conducted the FOCUS (S-1 Plus Oxaliplatin Compared With Fluorouracil, Leucovorin Calcium Plus Oxaliplatin as Perioperative Chemotherapy for Advanced Gastric Carcinoma) trial to compare the efficacy and safety of SOX with fluorouracil, leucovorin, and oxaliplatin (FOLFOX) as a perioperative chemotherapy regimen for locally advanced, resectable gastric cancer.

## Methods

### Study Design and Participants

The FOCUS trial was an investigator-initiated, multicenter, open-label RCT. We recruited patients from 12 Chinese hospitals. The inclusion criteria were as follows. First, patients had to have histopathologically confirmed gastric adenocarcinoma before randomization. Second, the primary tumor penetrated the serosa or invaded adjacent structures with or without metastatic lymph nodes (T4N) according to the American Joint Committee on Cancer and Union for International Cancer Control TNM classification for carcinoma of the stomach (7th edition).^11^ Staging investigation included endoscopy, computed tomography, endoscopic ultrasonography, and/or magnetic resonance imaging, according to local practice. The clinical stage was decided by an upper gastrointestinal multidisciplinary team. Third, patients were ambulatory men or women. Fourth, patients were aged 18 to 80 years old. Fifth, patients had Eastern Cooperative Oncology Group (ECOG) performance status scores of 0 to 2. Sixth, life expectancy was more than 3 months. Seventh, patients had sufficient bone marrow function (white blood cell count, 3500-12 000 cells/μL [to convert to cells × 10^9^/L, multiply by .001]; platelet count >100 × 10^3^/μL [to convert to cells × 10^9^/L, multiply by 1.0]), liver function (total bilirubin <1.5 times the upper limit of normal; alanine aminotransferase or aspartate aminotransferase <2.5 times the upper limit of normal), kidney function (calculated glomerular filtration rate >80 mL/min/1.73 m^2^ or serum creatinine <1.5 times the upper limit of normal), and cardiac function (ejection fraction >50% by echocardiography). Patients were excluded from the study if they had 1 of the following criteria: (1) distant metastasis according to the *American Joint Committee on Cancer Cancer Staging Manual* (7th edition)^11^; (2) history of major stomach surgery; (3) previous cytotoxic chemotherapy, radiotherapy, target therapy, or immunotherapy for any tumor; (4) history of another malignant neoplasm except for cured basal cell carcinoma of skin and cured carcinoma in situ of uterine cervix; (5) massive gastrointestinal hemorrhage and/or gastric outlet obstruction caused by tumor; (6) continuous systematic administration of corticosteroids; or (7) history of angina, myocardial infarction within 6 months or other serious uncontrolled concomitant diseases.

The study protocol was approved by the ethics committees of each participating institution. The study was done according to the Declaration of Helsinki^12^ and Good Clinical Practice Guidelines as defined by the International Conference on Harmonization.^13^ All enrolled patients provided written informed consent. The full trial protocol is available in Supplement 1. This study followed the Consolidated Standards of Reporting Trials (CONSORT) reporting guideline.

### Randomization

Eligible patients were randomly assigned (1:1) to receive either perioperative FOLFOX or SOX, in addition to surgery. The randomization sequence was generated by a statistician using SAS statistical software version 9.1 (SAS Institute). Every patient had a unique identification number and the allocation results were sent to investigators. Patients and investigators were not masked to the treatment allocation because the study was open label.

### Treatment

A total of 6 cycles of perioperative chemotherapy were scheduled for enrolled patients. SOX or FOLFOX was administered for 2 to 4 preoperative cycles followed by 2 to 4 postoperative cycles. Postoperative chemotherapy was to be started within 4 to 6 weeks after surgery. In the SOX group, oxaliplatin was administered as a 2-hour intravenous infusion (130 mg/m^2^) on day 1, and S-1 was given orally twice daily for 2 weeks followed by a 7-day rest period. The dose of S-1 was 80 mg/day for body surface area less than 1.25 m^2^, 100 mg/day for body surface area greater than or equal to 1.25 to less than 1.5 m^2^, and 120 mg/day for body surface area greater than or equal to 1.5 m^2^. The FOLFOX group had a regimen that consisted of oxaliplatin (130 mg/m^2^) as a 2-hour intravenous infusion on day 1, leucovorin (400 mg/m^2^), and a bolus of fluorouracil (400 mg/m^2^) on day 1, followed by a 46-hour infusion of fluorouracil (2400 mg/m^2^). Both regimens were repeated every 3 weeks. Adverse events during chemotherapy were graded according to National Cancer Institute Common Terminology Criteria for Adverse Events 3.0. Medications and supportive care were given to reduce the toxic effects according to the study protocol. If patients had grade 2 or grade 3 hematological toxic effects, or nonhematological toxic effect of grade 2, grade 3, or grade 4, S-1 was reduced by 1 dose level (from 120 mg to 100 mg, from 100 mg to 80 mg, or from 80 mg to 50 mg), and the dose of fluorouracil was reduced by 75% or to 50% if toxic effects recurred after the first dose reduction. The dose of oxaliplatin was adjusted for sensory peripheral neuropathy as per the study protocol. Chemotherapy was stopped for unacceptable toxic effects, disease progression, at the patient’s request, or if an investigator judged that the patient could benefit from stopping treatment. At baseline assessment and before the initiation of every cycle, enrolled patients in both groups were evaluated by medical history, physical examination, ECOG performance status, and hematological tests.

### Surgery

Surgery was scheduled within 2 weeks after completion of the last cycle of preoperative chemotherapy. Distal or total gastrectomy was conducted depending on the location and extent of the primary tumor. En bloc resection was performed if the tumor invaded the surrounding organs or structures. D2 lymphadenectomy (ie, resection of the regional lymph nodes, including the lymph nodes along right and left cardiac artery, lesser and greater curvature, suprapyloric along the right gastric artery, infrapyloric area, left gastric artery, common hepatic artery, celiac artery, splenic hilum, and splenic artery) was performed by experienced surgeons according to the criteria established by Japanese Gastric Cancer Association.^14^ Experienced surgeons who had performed at least 50 subtotal or total gastrectomies with D2 lymphadenectomy for patients with advanced gastric cancer annually participated in the trial. Any postoperative complications were evaluated within 30 days after surgery and graded using the Clavien-Dindo criteria.^15^ Pathological TNM staging was evaluated according to the 7th edition of TNM classification for gastric cancer.^11^

### Follow-up

After completion of the treatment, enrolled patients were followed up every 3 to 6 months from 1 to 2 years, 6 to 12 months from 3 to 5 years, and then annually after 5 years. The follow-up included complete blood counts, chemistry profile, tumor markers, and radiological examinations. Gastrointestinal endoscopy was conducted annually. Whether the tumor recurred or progressed was determined by radiological findings or tissue biopsy, if it was feasible. Cause of death and sites of recurrence or progression were assessed and recorded by investigators.

### Statistical Analysis

This was a multicenter, open-label, RCT that was planned to show the noninferiority of perioperative chemotherapy of SOX compared with FOLFOX. The primary end point was OS, which was defined as the time from the randomization to death of any cause. The secondary end point was progression-free survival (PFS), which was defined as the time from randomization until 1 of the following events had occurred: local progression, local recurrence, distant metastasis, or death from any cause. The assumed 3-year OS of patients with advanced gastric cancer who received perioperative chemotherapy of FOLFOX was approximately 32%, and the prespecified noninferiority margin was −8% for SOX. With a 10% dropout rate, 583 patients were required to reach a power of 80%, at a 2-sided type I error of .05. The OS and PFS were estimated by Kaplan-Meier analysis. To demonstrate noninferiority, we calculated the difference in 3-year OS between the 2 groups at 1-sided significance level of .025 and reported with a 2-sided 95% CI, using the Kaplan-Meier estimates and Greenwood estimates of the corresponding variance. We then established whether the difference was greater than the prespecified noninferiority margin of −8%.

OS and PFS were compared with the log-rank test, and hazard ratios (HRs) were calculated with a Cox proportional-hazards model after adjustment for all baseline stratification factors (including age, sex, ECOG performance status, and primary tumor location). The subgroup analysis was presented as a forest plot. Patients who completed at least 1 cycle of preoperative chemotherapy were evaluated for the safety of chemotherapy. Categorical variables were analyzed by Pearson χ^2^ test and, if necessary, by Fisher exact test. Continuous variables were compared by *t* test and, if necessary, by the Mann-Whitney *U *test. All statistical tests were 2-sided, and *P* < .05 indicated significance. All statistical analyses were done with R statistical software version 3.5.1 (R Project for Statistical Computing). Data were analyzed from December 2019 to June 2020.

## Results

### Patients

**Figure 1.**
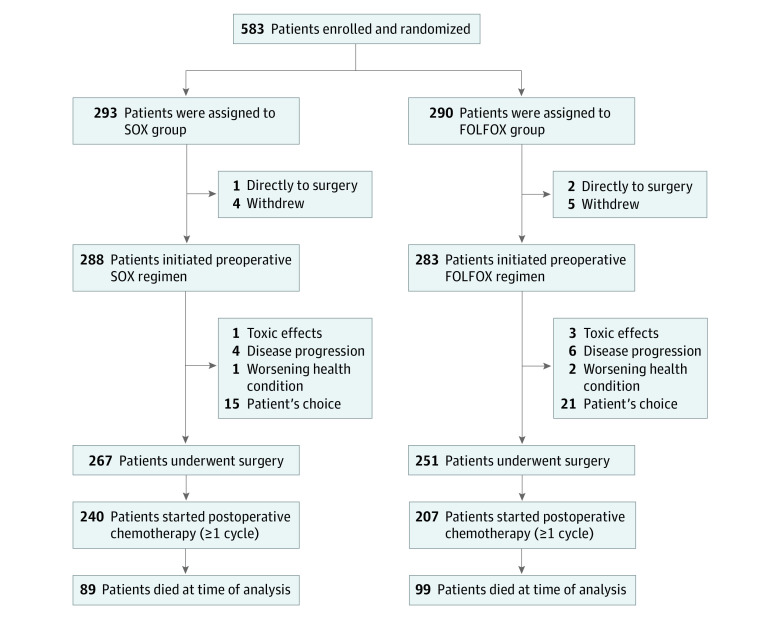
Trial Enrollment Flowchart FOLFOX indicates fluorouracil, leucovorin, and oxaliplatin; SOX, S-1 plus oxaliplatin.

Between June 2011 and August 2016, a total of 583 patients were enrolled; 293 patients were randomized to the SOX group and 290 patients were randomized to the FOLFOX group. With a last follow-up date of September 2019, 12 of 583 patients (2.1%) refused preoperative chemotherapy (5 patients in the SOX group and 7 patients in the FOLFOX group); therefore, 288 patients in the SOX group (median [range] age, 61 [24 to 78] years; 197 men [68.4%]) and 283 patients in the FOLFOX group (median [range] age, 62 [24 to 80] years; 209 men [73.9%]) received preoperative chemotherapy. The baseline characteristics (including age, sex, ECOG performance status, and primary tumor location) were well balanced between the 2 groups (Table 1). After the preoperative chemotherapy, 267 of 288 patients (92.7%) in the SOX group and 251 of 283 patients (88.7%) in the FOLFOX group proceeded to surgery. After surgery, 240 of 288 patients (83.3%) in the SOX group and 207 of 283 patients (73.1%) in the FOLFOX group received postoperative chemotherapy. Finally, 195 of 288 patients (67.7%) in the SOX group and 168 of 283 patients (59.4%) in the FOLFOX group completed all 6 cycles of perioperative chemotherapy (χ^2^_1_ = 4.29; *P* = .04). Figure 1 shows the trial profile and the reasons for patients who discontinued the protocol.

**Table 1.** Baseline Characteristics

Characteristic	Patients, No. (%)	
	SOX (n = 288)	FOLFOX (n = 283)
Age, y		
Median (range)	61 (24-78)	62 (24-80)
<60	120 (41.7)	120 (42.4)
≥60	168 (58.3)	163 (57.6)
Sex		
Male	197 (68.4)	209 (73.9)
Female	91 (31.6)	74 (26.1)
Eastern Cooperative Oncology Group status		
0	155 (53.8)	146 (51.6)
1	123 (42.7)	132 (46.6)
2	10 (3.5)	5 (1.8)
Primary tumor location		
Upper	59 (20.5)	40 (14.1)
Middle	58 (20.1)	65 (23.0)
Lower	152 (52.8)	160 (56.5)
Diffuse type	19 (6.6)	18 (6.4)

Abbreviations: FOLFOX, fluorouracil, leucovorin and oxaliplatin; SOX, S-1 and oxaliplatin.

### Chemotherapy-Associated Toxic Effects

The chemotherapy-associated toxic effects during perioperative chemotherapy are shown in the eTable in Supplement 2. The most common grade 3 or 4 hematological toxic effect was neutrocytopenia in both groups (56 of 288 patients [19.4%] in the SOX group and 69 of 283 patients [24.3%] in the FOLFOX group). However, we observed significantly more instances of grade 3 or 4 thrombocytopenia in the SOX group (32 of 288 patients [11.1%] in the SOX group vs 8 of 283 patients [2.8%] in the FOLFOX group; χ^2^_1_ = 15.04; *P* < .001). The most common grade 3 or 4 nonhematological toxic effect in SOX group was nausea (23 of 288 patients [8.0%]), followed by vomiting (20 of 288 patients [6.9%]). The most common grade 3 or 4 nonhematological toxic effect in the FOLFOX group was vomiting (28 of 283 patients [9.9%]), followed by nausea (18 of 283 patients [6.4%]). There was no significant difference between the 2 groups.

### Surgical and Pathological Findings

The surgical findings are shown in Table 2. There was no significant difference with respect of the type of gastrectomy and anastomosis between the 2 groups. D2 lymphadenectomy was done in 263 of 267 patients (98.5%) in the SOX group and 246 of 251 patients (98.0%) in the FOLFOX group. The number of dissected lymph nodes was not significantly different between the 2 groups (mean [SD], 35.4 [14.9] nodes in the SOX group and 33.6 [14.2] nodes in the FOLFOX group). R0 resection was similar between the 2 groups (255 of 267 patients [95.5%] in the SOX group and 240 of 253 patients [95.6%] in the FOLFOX group). The postoperative morbidity was also similar between the 2 groups (75 of 267 patients [28.1%] in the SOX group vs 56 of 251 patients [22.3%] in the FOLFOX group). The incidence of postoperative bleeding (8 of 267 patients [3.0%] in the SOX group vs 7 of 251 patients [2.8%] in the FOLFOX group) and that oof anastomotic leakage (1 of 267 patients [0.4%] in the SOX group vs 0 in the FOLFOX group) were comparable between 2 groups. One patient in the SOX group underwent reoperation because of anastomotic bleeding, and 2 patients in the FOLFOX group underwent reoperation because of intra-abdominal bleeding. No postoperative mortality within 30 days was observed in either group.

**Table 2.** Surgical and Pathological Findings

Findings	Patients, No./total No. (%)	
	SOX (n = 288)	FOLFOX (n = 283)
Proceeded to surgery	267/288 (92.7)	251/283 (88.7)
Type of gastrectomy		
Distal	139 /267 (52.1)	147/251 (58.6)
Total	126/267 (47.2)	101/251 (40.2)
Unknown	2/267 (0.7)	3/251 (1.2)
Combined resection	8/267 (3.0)	7/251 (2.8)
Lymphadenectomy		
D1	1 /267(0.4)	2/251 (0.8)
D2	263/267 (98.5)	246/251 (98.0)
Unknown	3/267 (1.1)	3/251 (1.2)
Type of anastomosis		
Billroth-I	6/267 (2.2)	8/251 (3.2)
Billroth-II	131/267 (49.1)	137/251 (54.6)
Roux-en-Y	128/267 (47.9)	103/251 (41.0)
Unknown	2/267 (0.7)	3/251 (1.2)
Tumor residual		
R0	255/267 (95.5)	240/251 (95.6)
R1	3/267 (1.1)	2/251 (0.8)
R2	7/267 (2.6)	6/251 (2.4)
Unknown	2/267 (0.7)	3/251 (1.2)
Pathological findings		
Tumor stage (ypT)		
T0	11/267 (4.1)	5/251 (2.0)
T1	43/267 (16.1)	26/251 (10.4)
T2	35/267 (13.1)	35/251 (13.9)
T3	18/267 (6.7)	11/251 (4.4)
T4	148/267 (55.4)	169/251 (67.3)
Tx	10/267 (3.7)	2/251 (0.8)
Unknown	2/267 (0.7)	3/251 (1.2)
Nodal status (ypN)		
N0	118/267 (44.2)	93/251 (37.1)
N1	55/267 (20.6)	48/251 (19.1)
N2	39/267 (14.6)	64/251 (25.5)
N3	52/267 (19.5)	43/251 (17.1)
Unknown	3/267 (1.1)	3/251 (1.2)
Distant metastasis (ypM)		
M0	259/267 (97.0)	239/251 (95.2)
M1	6/267 (2.2)	9/251 (3.6)
Unknown	2/267 (0.7)	3/251 (1.2)

Abbreviations: FOLFOX, fluorouracil, leucovorin and oxaliplatin; SOX, S-1 and oxaliplatin.

The postoperative pathological findings are summarized here. Nine of 267 patients (3.4%) in the SOX group and 4 of 251 patients (1.6%) in the FOLFOX group achieved pathological complete response after preoperative chemotherapy. The ypT stage could not be classified in 12 patients (10 of 267 patients [3.7%] in the SOX group and 2 of 251 patients [0.8%] in the FOLFOX group), because of only single cells or rare small groups of cancer cells were residual. There was a lower proportion of ypT4 stage disease in the SOX group than in the FOLFOX group (148 of 267 patients [55.4%] vs 169 of 251 patients [67.3%]; χ^2^_1_ = 7.71; *P* = .005). However, the stage ypN0 disease was not significantly different between the 2 groups (118 of 267 patients [44.2%] in the SOX group vs 93 of 251 patients [37.1%] in the FOLFOX group).

### Survival Analysis

**Figure 2.**
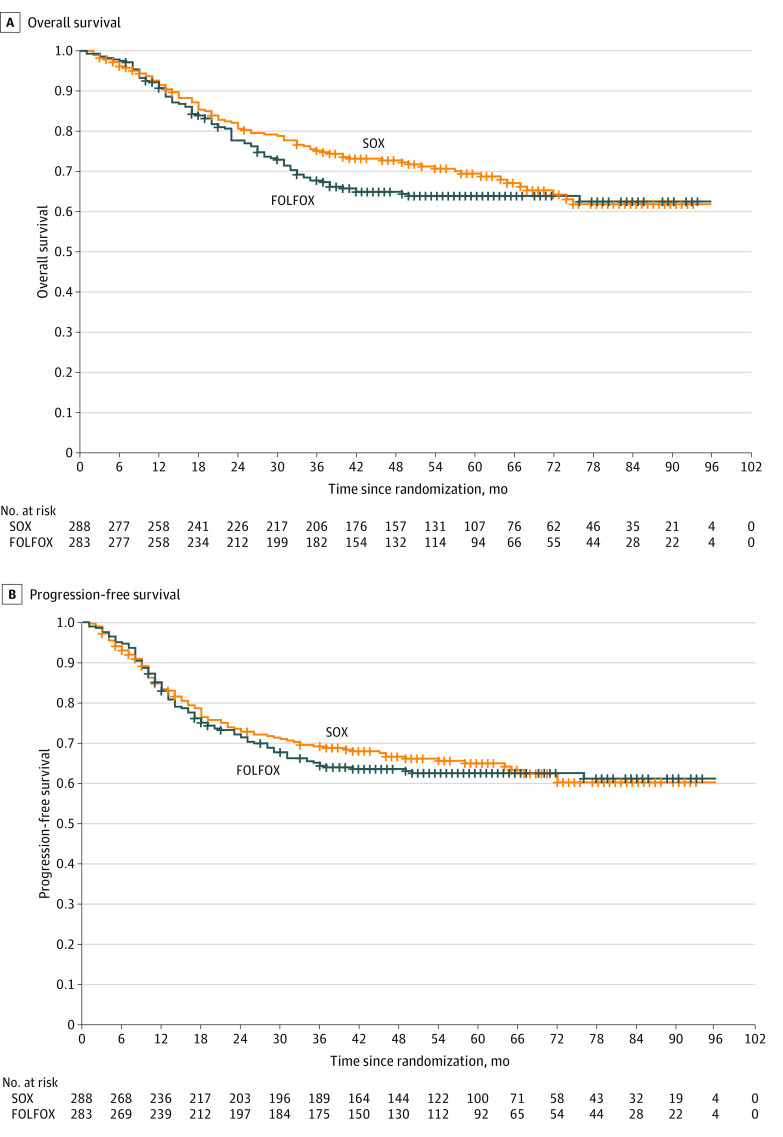
Kaplan-Meier Survival Estimates A, The 3-year overall survival rate was 75.2% for the S-1 plus oxaliplatin (SOX) group and 67.8% for the fluorouracil, leucovorin, and oxaliplatin (FOLFOX) group (hazard ratio, 0.84; 95% CI, 0.63-1.13). B, The 3-year progression-free survival rate was 69.3% for the SOX group and 64.5% for the FOLFOX group (HR, 0.95; 95% CI, 0.72-1.25).

**Figure 3.**
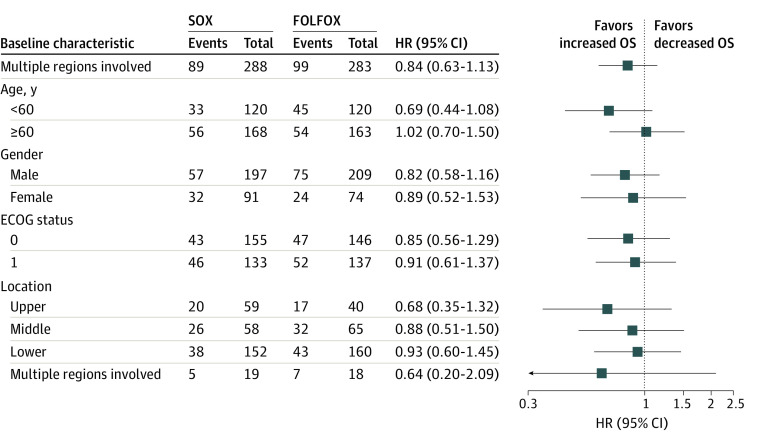
Subgroup Analysis of Overall Survival (OS) in the S-1 Plus Oxaliplatin (SOX) Group Compared With the Fluorouracil, Leucovorin, and Oxaliplatin (FOLFOX) Group Hazard ratios (HRs) for death and *P* values show no significance of heterogeneity of treatment effect according to the sex, age, Eastern Cooperative Oncology Group (ECOG) status, and primary tumor location.

At the time of analysis, the median (range) follow-up time was 61 (2 to 96) months in the SOX group and 60 (1 to 96) months in the FOLFOX group. Local recurrence was confirmed in 11 of 288 patients (3.8%) in the SOX group and 7 of 283 patients (2.5%) in the FOLFOX group, and distant metastases were confirmed in 70 of 288 patients (24.3%) in the SOX group and 67 of 283 patients (23.6%) in the FOLFOX group. At analysis, 89 of 288 patients (30.9%) in the SOX group and 99 of 283 patients (35.0%) in the FOLFOX group had died. The OS and PFS are shown in Figure 2. The 3-year OS rates were 75.2% (95% CI, 70.3% to 80.5%) for the SOX group and 67.8% (95% CI, 62.5% to 73.5%) for the FOLFOX group (HR, 0.84; 95% CI, 0.63 to 1.13). The absolute difference between the 2 groups in 3-year OS rate was 7.4% (95% CI, –0.1% to 14.9%), which is greater than the prespecified noninferiority margin of –8%, demonstrating the noninferiority of SOX compared with FOLFOX therapy. Figure 3 shows no clear evidence of heterogeneity of treatment effect according to sex, age, ECOG performance status, and primary tumor location. The 3-year PFS rate was 69.3% (95% CI, 64.0% to 74.9%) for the SOX group and 64.5% (95% CI, 59.1% to 70.4%) for the FOLFOX group (HR, 0.95; 95% CI, 0.72 to 1.25).

## Discussion

In this RCT of patients with locally advanced gastric cancer, we found that the absolute difference of 3-year OS rate between the 2 groups was 7.4% (75.2% in the SOX group vs 67.8% in FOLFOX group). The lower limit of the 95% CI was –0.1%, which was greater than the prespecified noninferiority margin (–8%) and shows the noninferiority of perioperative chemotherapy with SOX compared with therapy with FOLFOX.

According to the results of several phase 3 studies,^4,5,16^ perioperative chemotherapy was recommended as an alternative treatment for patients with gastric cancer of stage T2 or higher. The landmark MAGIC trial demonstrated the survival benefit of perioperative chemotherapy with epirubicin, cisplatin, and fluorouracil compared with surgery alone in locally advanced gastric cancer patients.^4^ The subsequent FLOT4 trial showed that a FLOT regimen (fluorouracil plus leucovorin, oxaliplatin, and docetaxel) was superior to epirubicin and cisplatin plus either fluorouracil or capecitabine as perioperative chemotherapy.^16^ However, in Asia, both SOX and FOLFOX were found to be effective and well tolerated in patients with unresectable gastric cancer,^17,18^ but the role of these perioperative chemotherapy regimens for locally advanced gastric cancer was not fully investigated. This phase 3 RCT sought to analyze the outcome for these regimens for 583 patients with locally advanced gastric cancer, 571 of whom received chemotherapy and were included in the final analysis. The 3-year OS rates were 75.2% for the SOX group and 67.8% for the FOLFOX group. In comparison, the FLOT4 trial reported a 3-year OS rate of 57%.^16^ However, patients with stage II or higher disease were enrolled in the MAGIC trial,^4^ and the FLOT4 trial^16^ enrolled patients with clinical stage cT2 or higher or nodal positive stage (cN+), which represented the advanced gastric cancer. One of the advantages of preoperative chemotherapy is increasing the R0 resection rate. In China, patients with more advanced gastric cancer were selected. In our study, patients with cT4a disease or higher (the majority of cases were equivalent to pathological stage III) were enrolled irrespective of clinical N status. The high survival rate might be associated with the high rate of D2 lymphadenectomy (98.5% in the SOX group and 98.0% in the FOLFOX group) or the high percentage of patients who completed 6 cycles of perioperative chemotherapy (67.7% in the SOX group and 59.4% in the FOLFOX group). The better tolerance of the doublet regimens was considered to be associated with the higher percentage of patients receiving postoperative chemotherapy. In addition, more patients commenced postoperative chemotherapy and completed all 6 cycles of perioperative chemotherapy in the SOX group, which might be associated with the convenience of oral administration of S-1.^8^

Recently, the results of 2 phase 3 trials of perioperative chemotherapy have been reported (RESOLVE from China and PRODIGY from Korea). The supplemental data of the RESOLVE trial also showed that approximately one-third of patients had died at the time of analysis (109 of 337 patients)^19^; meanwhile, the 3-year OS was 74.2% in the PRODIGY trial.^20^ Our results were comparable to these 2 trials. With regard to PFS, the 3-year PFS reported in the PRODIGY trial was 74.2%, whereas in our study, the 3-year PFS was 69.3% in the SOX group and 64.5% in the FOLFOX group. The 3-year PFS in the RESOLVE trial was 59.4%, which was a little lower. We thought that this difference in findings might be related to the different populations, in that patients with cT4aN+ disease or patients with any cT4bN status were enrolled in RESOLVE.

In terms of adverse events, the toxic effect profiles were similar between the 2 groups, and both regimens showed acceptable tolerability. The most common toxic effects of both regimens in our study were leukocytopenia, neutrocytopenia, thrombocytopenia, and anemia. Thrombocytopenia was an important hematological toxic effect that might lead to the treatment delay and discontinuation. In previous studies,^21,22,23^ the reported incidence of grade 3 or 4 thrombocytopenia ranged from 2% to 5% with FOLFOX regimen in patients with unresectable or recurrent gastric cancer. In the present trial, the incidence of grade 3 or 4 thrombocytopenia in the FOLFOX group was 2.8%. However, the incidence of grade 3 or 4 thrombocytopenia was much higher in the SOX group than in the FOLFOX group (11.1% vs 2.8%; *P* < .001). The incidence of thrombocytopenia in the SOX group in the present study was in line with the results of previous studies.^17,24^ It was reported that the chemotherapy-induced sinusoidal obstruction syndrome was associated with thrombocytopenia.^25^ The sinusoidal obstruction syndrome was clinically characterized by portal hypertension, splenomegaly, subsequent thrombocytopenia, and liver dysfunction, and occurred frequently during oxaliplatin-based chemotherapy.^26,27,28^ Recently, one study^29^ found that SOX is more likely to worsen oxaliplatin-induced hepatic sinusoidal injuries than capecitabine and oxaliplatin in patients with gastric cancer, and those authors proposed that S-1 could potentiate the toxic effects of fluorouracil-induced hepatocellular inflammation by inhibiting fluorouracil degradation through its component gimeracil. However, the exact mechanism needs to be further investigated. With regard to nonhematological toxic effects, patients in the SOX group experienced fewer gastrointestinal toxic effects (eg, anorexia or nausea) than those in the FOLFOX group in the present study. This may be associated with the potassium oteracil in S-1, which could inhibit the phosphorylation of fluorouracil in the gastrointestinal tract, subsequently decreasing gastrointestinal toxic effects.

There was no significant difference in 30-day postoperative morbidity between the 2 groups (28.1% for the SOX group vs 22.3% for the FOLFOX group), and no patient died within 30 days after surgery. The incidence of postoperative bleeding (8 of 267 patients [3.0%] in SOX group, and 7 of 251 patients [2.8%] in FOLFOX group) and anastomotic leakage (1 of 267 patients [0.4%] in SOX group, and none in FOLFOX group) was comparable between the 2 groups. The low postoperative morbidity and mortality in the present study were consistent with those for patients in a previous study who underwent D2 lymphadenectomy in Asia,^30^ indicating that perioperative chemotherapy with SOX or FOLFOX was safe for patients with locally advanced gastric cancer.

### Limitations

The current study has some limitations. First, the duration of the study was more than 8 years, and the treatment strategy for recurrent or metastatic gastric cancer has significantly improved during this period. Furthermore, the psychological and treatment intentions of patients have also been enhanced since these data were collected. These factors may be partly associated with the high rate of 3-year OS in the present study. In addition, the open-label treatment may have caused information bias.

## Conclusions

In this RCT, SOX was noninferior to FOLFOX as perioperative chemotherapy for patients with locally advanced gastric cancer. These findings suggest that SOX could be recommended as an alternative perioperative chemotherapy for these patients in Asia.

## Notes

Supplement 1.
Trial Protocol and Statistical Analysis Plan

Supplement 2. **eTable.** Hematological and Nonhematological Toxicities Associated With Perioperative Chemotherapy

Supplement 3.
Data Sharing Statement
