# Supplementary material for: Effect of S-1 Plus Oxaliplatin Compared With Fluorouracil, Leucovorin Plus Oxaliplatin as Perioperative Chemotherapy for Locally Advanced, Resectable Gastric Cancer: A Randomized Clinical Trial
Source: JAMA Netw Open. 2022 Feb 28;5(2):e220426. doi: 10.1001/jamanetworkopen.2022.0426 (PMC8886520; doi:10.1001/jamanetworkopen.2022.0426)
Supplement: Supplement 2. — eTable. Hematological and Nonhematological Toxicities Associated With Perioperative Chemotherapy [file jamanetwopen-e220426-s002.pdf]

Supplemental Online Content

Yu J, Gao Y, Chen L, et al. Effect of S-1 plus oxaliplatin compared with fluorouracil, leucovorin plus oxaliplatin as perioperative chemotherapy for locally advanced, resectable gastric cancer: a randomized clinical trial. *JAMA Netw Open*. 2022;5(2):e220426. doi:10.1001/jamanetworkopen.2022.0426

eTable. Hematological and Nonhematological Toxicities Associated With Perioperative Chemotherapy

This supplemental material has been provided by the authors to give readers additional information about their work.

© 2022 Yu J et al. *JAMA Network Open.*

eTable. Hematological and Nonhematological Toxicities Associated With Perioperative Chemotherapy

	SOX (N=288)				FOLFOX (N=283)		
	All grades no. (%)	Grade 3/4 no. (%)	RR (95% CI)		All grades no. (%)	Grade 3/4 no. (%)	RR (95% CI)
**Hematologic**							
Leukocytopenia	218(75.7)	17(5.9)	1.12 (0.77, 1.61)		192(67.8)	12(4.2)	1.39 (0.65, 2.97)
Neutrocytopenia	224(77.8)	56(19.4)	1.04 (0.71, 1.53)		212(74.9)	69(24.3)	0.80 (0.54, 1.19)
Thrombocytopenia	173(60.1)	32(11.1)	1.87 (1.33, 2.63)		91(32.2)	8(2.8)	3.93 (1.78, 8.69)
Anemia	192(66.7)	33(11.5)	1.26 (0.90, 1.76)		150(53.0)	26(9.2)	1.25 (0.73, 2.15)
**Non-hematologic**							
ALT	140(48.6)	2(0.7)	0.99 (0.71, 1.37)		139(49.1)	6(2.1)	0.33 (0.07, 1.64)
AST	136(47.4)	1(0.3)	1.26 (0.90, 1.76)		106(37.5)	3(1.1)	0.33 (0.03, 3.17)
Cr	35(12.2)	0	1.07 (0.65, 1.79)		32(11.3)	0	NA
Fatigue	100(34.7)	3(1.0)	0.81 (0.58, 1.14)		121(42.8)	4(1.4)	0.74 (0.16, 3.32)
Alopecia	33(11.5)	NA	0.60 (0.38, 0.96)		54(19.1)	NA	NA
Hyperpigmentation	75(26.0)	NA	1.45 (0.97, 2.16)		51(18.0)	NA	NA
Fever	35(12.2)	1(0.3)	1.23 (0.73, 2.08)		28(9.9)	3(1.1)	0.33 (0.03, 3.17)

© 2022 Yu J et al. *JAMA Network Open.*

Hand-foot syndrome	40(13.9)	0	1.51 (0.90, 2.55)		26(9.2)	3(1.1)	NA
Anorexia	78(27.1)	1(0.3)	0.67 (0.47, 0.95)		115(40.6)	2(0.7)	0.49 (0.04, 5.45)
Constipation	34(11.8)	1(0.3)	0.98 (0.59, 1.63)		34(12.0)	0	NA
Diarrhea	75(26.0)	5(1.7)	1.10 (0.75, 1.61)		67(23.7)	4(1.4)	1.23 (0.33, 4.62)
Mucositis	40(13.9)	1(0.3)	0.98 (0.61, 1.58)		40(14.1)	2(0.7)	0.49 (0.04, 5.45)
Nausea	158(54.9)	23(8.0)	0.85 (0.61, 1.19)		183(64.7)	18(6.4)	1.26 (0.66, 2.38)
Vomiting	129(44.8)	20(6.9)	0.99 (0.71, 1.38)		128(45.2)	28(9.9)	0.70 (0.39, 1.28)
Sensory neuropathy	127(44.1)	2(0.7)	0.91 (0.66, 1.27)		137(48.4)	1(0.4)	1.97 (0.18, 21.80)

SOX=S-1 and oxaliplatin; FOLFOX=fluorouracil leucovorin and oxaliplatin; RR=relative risk; CI= confidence interval; ALT=alanine aminotransferase; AST=aspartate aminotransferase; Cr=Creatinine; NA=not applicable. Grade3/4 is not applicable for alopecia and hyperpigmentation according to Common Terminology Criteria for Adverse Events (CTCAE) 3.0.

© 2022 Yu J et al. *JAMA Network Open.*
