# Supplementary material for: Effect of S-1 Plus Oxaliplatin Compared With Fluorouracil, Leucovorin Plus Oxaliplatin as Perioperative Chemotherapy for Locally Advanced, Resectable Gastric Cancer: A Randomized Clinical Trial
Source: JAMA Netw Open. 2022 Feb 28;5(2):e220426. doi: 10.1001/jamanetworkopen.2022.0426 (PMC8886520; doi:10.1001/jamanetworkopen.2022.0426)
Supplement: Supplement 3. — Data Sharing Statement [file jamanetwopen-e220426-s003.pdf]

Data Sharing Statement

Yu. Effect of S-1 Plus Oxaliplatin Compared With Fluorouracil, Leucovorin Plus Oxaliplatin as Perioperative Chemotherapy for Locally Advanced, Resectable Gastric Cancer. *JAMA Netw Open*. Published February 28, 2022. doi:10.1001/jamanetworkopen.2022.0426

Data
Data available: Yes
Data types: Deidentified participant data
How to access data: yujr0909@zju.edu.cn
When available: With publication

Supporting Documents
Document types: None

Additional Information
Who can access the data: Anyone whose proposed use of the data has been approved
Types of analyses: for any purpose
Mechanisms of data availability: with investigator support, after approval of a proposal and with a signed data access agreement
Any additional restrictions: no
